# Cortactin expression predicts poor survival in laryngeal carcinoma

**DOI:** 10.1038/sj.bjc.6604246

**Published:** 2008-02-12

**Authors:** J H Gibcus, M F Mastik, L Menkema, G H de Bock, Ph M Kluin, Ed Schuuring, J E van der Wal

**Affiliations:** 1 Department of Pathology, University Medical Center Groningen, University of Groningen, Groningen, The Netherlands; 2 Department of Epidemiology and Statistics, University Medical Center Groningen, University of Groningen, Groningen, The Netherlands

**Keywords:** HNSCC, larynx, 11q13, cortactin, FADD, cyclin D1

## Abstract

Amplification of the 11q13 region is one of the most frequent aberrations in squamous cell carcinomas of the head and neck region (HNSCC). Amplification of 11q13 has been shown to correlate with the presence of lymph node metastases and decreased survival. The 11q13.3 amplicon carries numerous genes including cyclin D1 and cortactin. Recently, we reported that FADD becomes overexpressed upon amplification and that FADD protein expression predicts for lymph node positivity and disease-specific mortality. However, the gene within the 11q13.3 amplicon responsible for this correlation is yet to be identified. In this paper, we compared, using immunohistochemical analysis for cyclin D1, FADD and cortactin in a series of 106 laryngeal carcinomas which gene correlates best with lymph node metastases and increased disease-specific mortality. Univariate Cox regression analysis revealed that high expression of cyclin D1 (*P*=0.016), FADD (*P*=0.003) and cortactin (*P*=0.0006) predict for increased risk to disease-specific mortality. Multivariate Cox analysis revealed that only high cortactin expression correlates with disease-specific mortality independent of cyclin D1 and/or FADD. Of genes located in the 11q13 amplicon, cortactin expression is the best predictor for shorter disease-specific survival in late stage laryngeal carcinomas.

Current methods to predict the outcome of head and neck squamous cell carcinoma (HNSCC) patients mainly involve clinicopathological parameters such as tumour size, differentiation grade and presence of lymph node metastasis. Patients with laryngeal squamous cell carcinomas (LSCC) have not shown an increase in 5-year survival rates over the last 30 years (Almadori *et al*, 2005). Therefore, other parameters such as molecular markers that are able to more accurately predict the outcome of disease, are needed.

A frequent molecular event in HNSCC is amplification of the 11q13 region (36%) (Schuuring, 1995; Freier *et al*, 2006). Amplification of this region in HNSCC has been associated with decreased survival (Akervall *et al*, 1995; Meredith *et al*, 1995), increased distant metastasis (Namazie *et al*, 2002) and lymph node metastasis (Chen *et al*, 2004; Hermsen *et al*, 2005; Xia *et al*, 2007). It is believed that amplification increases gene dosage and expression of genes within the amplified region (amplicon) (Albertson, 2006). Recently, we reported that the commonly amplified region is located at 11q13.3 and contains at least six genes (cyclin D1, TAOS1, FGF19, FADD, PPFIA1 and cortactin) that are overexpressed when amplified (Gibcus *et al*, 2007b). We concluded that the selection for tumour cells with the 11q13.3 amplicon during tumorigenesis could be based on the increased doses of one or more of these genes. We proposed FADD as a possible candidate for this selection, since FADD showed the best correlation between DNA amplification and increased expression (Gibcus *et al*, 2007b). Furthermore, FADD protein expression correlated with disease specific mortality (DSM) in a series of late stage laryngeal carcinomas. FADD protein expression was independent of lymph node metastasis and patients with both high FADD protein expression and lymph node metastasis had the worst prognosis for survival (Gibcus *et al*, 2007b). However, although FADD is a prognostic marker for increased mortality, we can not exclude that other genes within the 11q13 region are (also) involved.

The most frequently studied genes within the 11q13.3 region are cyclin D1 and cortactin (Schuuring, 1995). Overexpression of cyclin D1 at both protein and RNA level have been linked to poor prognosis in numerous studies (Akervall *et al*, 1997; Yu *et al*, 2005; Higuchi *et al*, 2007). Cortactin amplification was related to increased lymph node stage, advanced disease stage and reduced survival (Rodrigo *et al*, 2000). Cortactin mRNA expression (Luo *et al*, 2006) and protein expression (Rothschild *et al*, 2006) have been correlated to increased lymph node metastasis.

In summary, FADD, cyclin D1 and cortactin expression may each be involved in processes resulting in increased lymph node metastasis and in poor prognosis. Because the prognostic value of these genes has not been evaluated within the same group of patients simultaneously, we have performed immunohistochemistry (IHC) on 167 patients with late stage LSCC. Since lymph node metastases have been shown to be an important prognostic factor for DSM, we assessed the additional prognostic value of cyclin D1, FADD and cortactin in late stage LSCC.

## MATERIALS AND METHODS

### Patient inclusion

For immunohistochemical analysis we selected 167 patients diagnosed with a squamous cell carcinoma of the larynx, previously used for IHC of FADD. Patient material and available clinico-pathological data were obtained from the University Medical Center Groningen (*n*=56), The Netherlands Cancer Institute–Antoni Van Leeuwenhoek Hospital (*n*=62), the Instituto Universitario de Oncología del Principado de Asturias (*n*=22) and the Leiden University Medical Center, Leiden, the Netherlands (*n*=28). Successful staining for all three antibodies was examined on 120 patients. Sufficient follow-up was available for 106 of 120 patients. In the other 61 cases follow-up was short or unavailable, material was lost in the IHC-process or, no or too few tumour cells were present in the tissue block. The remaining patient group (*n*=106) consisted mainly of larger tumours: T1 (*n*=8; 8%), T2 (*n*=22; 21%), T3 (*n*=31; 29%) and T4 (*n*=45; 42%) that were either treated with surgery, radiotherapy or a combination of both treatments. Patients who did not die had a median follow-up of 72 months, whereas patients who died had a median follow-up of 16 months. The clinical characteristics of the 106 cases were comparable to the characteristics of the total group of 167 patients (see Table 1).

### Immunohistochemistry

Immunohistochemistry for cyclin D1 was performed as previously described (de Boer *et al*, 1995; Takes *et al*, 1998). For the immunostaining of cortactin we used a commercially available antibody (clone 30/cortactin from BD Transduction Laboratories, Franklin Lakes, NJ, USA) that recognizes all cortactin isoforms and can be applied on archival material. IHC for FADD on the larger series of 167 LSCC was reported earlier (Gibcus *et al*, 2007b). Ki67 expression was used as a marker for cell proliferation. Paraffin-embedded, formalin-fixed sections of laryngeal carcinoma were deparaffinized and antigen retrieval was performed by overnight incubation at 80°C in Tris–HCL pH=9.0 for Ki-67 and FADD, or heating in a microwave oven for 15 min in EDTA pH=6.0 for cortactin, or heating in a microwave oven in Tris–HCL pH=9.0 for 30 min for cyclin D1. After blocking endogenous peroxidases with 0.3% H_2_O_2_, the sections were stained for 1 h with an antibody against cyclin D1 (sp4, 1 : 50, Abcam, Cambridge, UK), FADD (1 : 100, BD Transduction Laboratories), cortactin (clone 30/cortactin; 1 : 1000, BD Transduction Laboratories) and Ki-67 (MIB-1, 1 : 350; DAKO, Heverlee, Belgium). Secondary and tertiary antibodies were diluted 1 : 100 in 1% BSA-PBS complemented with 1% AB-serum or EnVision (DAKO). Antibodies were precipitated using 3, 3′ diaminobenzidine tetrachloride as a substrate and the slides were counterstained using routine haematoxylin treatment. In parallel, a haematoxylin and eosin stained slide was scored by a pathologist (JEvdW) to verify tumour content.

Scoring of the immunohistochemical staining of Cyclin D1 and ki-67 was based on the percentage of positive tumour cells as reported previously. Scoring for cortactin was also based on the percentage of cells with high cytoplasmic expression. Expression levels similar or lower than the intensity in the surrounding normal tissue (used as the internal reference for staining) were considered as low cortactin expressors and those with increased intensity as high expressors. The intermingled lymphocytes served as a negative control. For FADD the intensity of the positively stained tumour cells was scored and divided into two different categories; low expressors (−, +) and high expressors (++, +++) as described previously (Gibcus *et al*, 2007b).

Antibodies against PPFIA1 (LIP.1) (Serra-Pages *et al*, 1995) and FGF19 (mab969; R&D Systems, Minneapolis, USA) appeared to be inappropriate for IHC analysis (data not shown) and antibodies against FLJ42258 and TAOS1 are presently not available.

### Statistical analysis

Statistical analysis was performed using version 14.0 of the SPSS software package. Cutoff percentages for dichotomization of the data were determined for cyclin D1 and cortactin using the median percentage of stained cells; 23% for nuclear cyclin D1 and 13% for cytoplasmic cortactin staining (Table 1). Associations between IHC staining patterns were identified by logistic regression. Prognostic value was evaluated per staining using Kaplan–Meier curves and univariate Cox regression for DSM to discern between deaths without evidence of disease and death of disease. A multivariate Cox regression model including cyclin D1, FADD, cortactin and lymph node status and treatment were based on univariate significance. All tests were two-sided and *P*-values of <0.05 were considered significant.

## RESULTS

**Figure 1:**
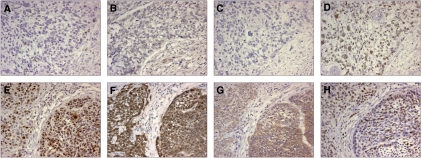
Immunohistochemical staining for cyclin D1, cortactin and FADD. A case without amplification and low cyclin D1 (**A**), FADD (**B**) and cortactin (**C**) expression and a case with amplification and high expression of cyclin D1 (**E**), FADD (**F**) and cortactin (**G**). Expression of ki-67 was unrelated to expression of the 11q13.3 genes (**D**, **H**).

To study which gene within the 11q13. 3 region is responsible for the clinical outcome, we evaluated the expression of cyclin D1, FADD and cortactin using a series of 106 LSSCs previously stained for FADD expression (Table 1 and Figure 1). The other four consistently overexpressed genes in the 11q13 amplicon (Gibcus *et al*, 2007b) were not investigated further because no antibodies were available (TAOS1 and FLJ42258) or appeared to be inappropriate for immunohistochemistry on archive material (PPFIA1 and FGF19) (data not shown). Firstly, we determined whether there was a link between amplification and protein expression. For this purpose we immunostained a subset of 16 cases in which amplification was determined previously (Gibcus *et al*, 2007a). Carcinomas with 11q13 amplification showed high protein expression levels in 8/8 cases for cyclin D1, 8/9 cases for FADD and 8/9 for cortactin (Table 2). In addition, the high expression of cyclin D1 and cortactin in 5/8 and 3/7 cases without amplification, respectively, and FADD only in 1/7 suggest that FADD correlates best with amplification (Table 2). The frequencies of high expression without amplification indicate that increased expression of cyclin D1 and cortactin are caused by mechanisms other than amplification.

Secondly, we evaluated whether protein expression of FADD, cyclin D1 and cortactin (Figure 1) were related to increased DSM and lymph node metastasis. Logistic regression for expression of FADD, cyclin D1 and cortactin was performed on dichotomized groups. The resulting odds ratios (OR) implicated that cortactin positivity (OR=11.2; 95% CI, 4.6–27.1) and cyclin D1 positivity (OR=3.82; 95% CI, 1.7–8.4) were related to FADD expression (data not shown). The relation between protein expression and increased DSM was determined using univariate Cox regression. High expression of FADD, cyclin D1 and cortactin was related to increased DSM (Table 3). Furthermore, lymph node metastasis, a marker for worse prognosis, was also related to increased DSM, whereas age, gender and tumour size were not. Treatment was also related to increased DSM. However, the choice of treatment is based on the prognosis obtained by classical prognostic factors (such as lymph node metastasis). Cell division rates, determined by immunohistochemical staining for Ki-67 levels (Figure 1D and H), were not correlated to increased DSM in univariate Cox regression. Although lymph node metastasis has been reported as an important clinical prognosticator (Eiband *et al*, 1989; Gasparotto and Maestro, 2007), cortactin expression (HR=13.14; 95% CI, 3.04–56.87; *P*=0.0006) correlated stronger to increased DSM than lymph node metastasis (HR=3.16; 95% CI, 1.29–7.75; *P*=0.012) (Table 3).

**Figure 2:**
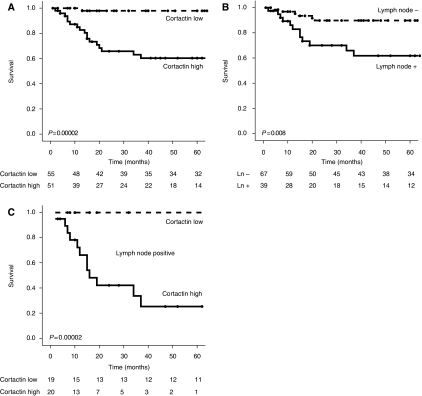
Kaplan–Meier analysis on disease-specific mortality for cortactin (**A**), lymph node positivity (**B**) and cortactin positivity within lymph node-positive cases (**C**). Remaining cases are shown below the plots.

A multivariate Cox regression model including cortactin, cyclin D1, FADD, treatment and lymph node metastasis showed that cortactin expression (HR=8.46; 95% CI, 1.63–43.88; *P*=0.011) and lymph node metastasis (HR=3.96; 95% CI, 1.30–12.06; *P*=0.015) were predictors for increased DSM, whereas FADD and cyclin D1 were not (Table 4A). Furthermore, in the multivariate analysis treatment with surgery, radiotherapy or a combination of both was not related to increased DSM (Table 4A). To assess whether cortactin and lymph node metastasis were independent prognostic factors, a multivariate Cox regression, using only cortactin expression and lymph node metastasis, was performed. Both cortactin expression and lymph node metastasis were significantly related to increased DSM (Table 4B), implying they are both prognostic factors. However, Kaplan–Meier analysis for cortactin, within lymph node positive tumours only, revealed a remarkable difference (Log-rank: *P*=0.00002) in DSM between cortactin-positive and cortactin-negative cases (Figure 2).

## DISCUSSION

### Cortactin predicts poor survival in late stage laryngeal carcinomas

Amplification of the 11q13.3 region has been related to a worse prognosis (Meredith *et al*, 1995; Xie *et al*, 2006). Furthermore, expression of cortactin, cyclin D1 and more recently FADD (Gibcus *et al*, 2007b), have been described separately as potential predictors for increased disease-related mortality, for lymph node metastasis and poor prognosis. Yet, the effect of overexpression due to amplification in head and neck carcinomas have not been examined for these proteins within the same group of patients.

We show that both cortactin and lymph node metastasis are good predictors for DSM using a multivariate model independent of cyclin D1 and FADD (Table 4A). This implies a correlation between lymph node metastasis and cortactin, as suggested previously (Rothschild *et al*, 2006). However, also within the lymph node metastasis positive group, high cortactin expression identifies patients with significantly increased DSM, whereas low cortactin expression identifies a group of patients with a good prognosis for survival. These data indicate that cortactin expression is independent of lymph node metastasis (Figure 2).

Cortactin, a regulator of ARP2/3-mediated actin polymerization, is known to contribute to tumour cell growth and cancer progression (Weed and Parsons, 2001). Downregulation of cortactin in cancer cell lines decreases the cellular motility and ability to migrate (Rothschild *et al*, 2006; van Rossum *et al*, 2006) whereas overexpression resulted in an increased invasive potential (van Rossum *et al*, 2003; Rothschild *et al*, 2006). In addition to the effect on cell migration by mediating actin polymerization and cell adhesion (van Rossum *et al*, 2006), cortactin might also influence cell migration and metastasis by anoikis resistance and PI3K/Akt signalling (Luo *et al*, 2006; Luo and Wang, 2007). Because of these biological functions and its overexpression due to DNA amplification, cortactin is the most likely gene within the 11q13.3 amplicon to mediate the worse clinical behaviour of late stage LSCC with 11q13 amplification.

### A role for multiple genes in the biology of 11q13.3 amplification

To evaluate the significance of 11q13.3 amplification it is imperative to discern the genes selected upon by amplification based overexpression (‘drivers’) from those whose overexpression is apparent, yet unrelated to tumour progression (‘hitchhikers’). Gene by gene functional analyses have revealed that cyclin D1 (Opitz *et al*, 2001; Nelsen *et al*, 2005), FADD (Alappat *et al*, 2003; Chen *et al*, 2005) and cortactin (van Rossum *et al*, 2003; Luo *et al*, 2006) are potentially able to enhance cancer development. However, these studies do not account for the effect of multiple genes located within the 11q13.3 amplicon. By revealing a relationship to prognosis, we show that cortactin is a potential driver, independent of cyclin D1 and FADD. Cyclin D1 and FADD were not significantly related to short DSM in a multivariate Cox regression model in the present study. However, our present study contains mainly high T-stage tumour and the univariate significance of cyclin D1 and FADD implies a link to amplification. The 11q13.3 amplicon is 1.7 Mb in size and contains 13 genes in almost all HNSCC cases with 11q13.3 amplification (Gibcus *et al*, 2007b). Therefore, multiple genes are enabled to increase their copy number and expression by 11q13.3 amplification (Freier *et al*, 2006; Gibcus *et al*, 2007a). Considering the different functions of cortactin, cyclin D1 and FADD it would be interesting to see whether these genes, coamplified in the same tumour, have a function at specific stages of tumorigenesis. Increased expression of cyclin D1 is detected at early stages of tumorigenesis and found prior to amplification (Izzo *et al*, 1998). Interestingly, cyclin D1 overexpression enhances cell division and results in genomic instability (Nelsen *et al*, 2005). On the other hand, cortactin overexpression might induce an increase in the invasive potential affecting later stages of cancer development (this study). Furthermore, cortactin overexpression was reported to inhibit the ubiquitination-mediated degradation of the epidermal growth factor receptor, resulting in a sustained ligand-induced epidermal growth factor receptor activity (van Rossum *et al*, 2005). Overexpression promoted resistance to the EGFR kinase inhibitor gefitinib (Timpson *et al*, 2007) indicating that cortactin not only affects invasive but also therapeutic responsive properties of HNSCC cancers. Finally, FADD expression and phosphorylation have been shown to affect cell cycle progression (Alappat *et al*, 2003) and resistance to chemotherapy (Shimada *et al*, 2004). Moreover, treatment with combinations of chemotherapy and radiation (Forastiere *et al*, 2003; Vermorken *et al*, 2007) might be affected by FADD expression due to 11q13 amplification, as hypothesized previously (Gibcus *et al*, 2007b). Because amplification was proven an early event in tumorigenesis (Izzo *et al*, 1998; Burnworth *et al*, 2006), and amplicons are inherited during tumorigenesis, we hypothesize that the high occurrence of 11q13.3 amplification enables multiple genes to be beneficial to tumour progression at distinct stages of tumour development.

In summary, our data indicate that cortactin is a good predictor for disease-related mortality in late stage LSCC. Furthermore, within lymph node positive tumours, cortactin expression can be used to distinguish between a good and a bad prognosis.

## Figures and Tables

**Table 1:** Patient characteristics as determined for the entire series of 167 patients (series) and the overlapping group of 106 patients (overlap)

**Characteristic**	**Series (%)**	**Overlap (%)**
*Age*	155	106
Median	61	61
Range	34–89	34–89
<61	75 (48)	53 (50)
>=61	80 (52)	53 (50)
		
*Gender*	156	106
Male	136 (87)	91 (86)
Female	20 (13)	15 (14)
		
*T-status*	167	106
T1	22 (13)	8 (8)
T2	29 (17)	22 (21)
T3	40 (24)	31 (29)
T4	76 (46)	45 (42)
		
*Lymph nodes (cN)*	166	106
N0	102 (61)	67 (63)
N1	24 (15)	16 (15)
N2	35 (21)	21 (20)
N3	5 (3)	2 (2)
Negative (N−)	102 (61)	67 (63)
Positive (N+)	64 (39)	39 (37)
		
*Grade*	156	106
Well	37 (24)	25 (24)
Moderate	79 (51)	51 (48)
Poor	40 (26)	30 (27)
		
*Therapy*	143	106
Surgery	55 (38)	42 (40)
RT	24 (17)	16 (15)
Surgery + RT	64 (45)	48 (45)
		
*CCND1*	138	106
Median percentage	20%	23%
<23%	71 (51)	52 (49)
>23%	67 (49)	54 (51)
		
*CTTN*	136	106
Median percentage	10%	13%
<13%	71 (52)	55 (52)
>13%	65 (48)	51 (48)
		
*FADD*	140	106
−/+	78 (56)	60 (57)
++/+++	62 (44)	46 (43)

Abbreviation: RT=radiotherapy.

**Table 2:** Cross table showing the relation between amplification and immunohistochemical staining for cyclin D1, FADD and cortactin

	**Immunohistochemistry**		
	**Negative**	**Positive**	**Total**
*CCND1 amplification*			
Negative	3	5	8
Positive	0	8	8
			
*FADD amplification*			
Negative	6	1	7
Positive	1	8	9
			
*CTTN amplification*			
Negative	4	3	7
Positive	1	8	9

Pearson *χ*^2^ *P*-values for cyclin D1, FADD and cortactin were 0.055, 0.003 and 0.049 respectively.

**Table 3:** Univariate Cox regression for FADD, CCND1and CTTN to DSM

**Cox regression**		**Hazard ratio for DSM**	
**univariate**	***N* (%)**	**HR**	**95% CI**
*CCND1*	106		
Low (<23%)	52 (49)	1	
High (>23%)	54 (51)	3.51	1.27–9.70 (*P*=0.016)
			
*FADD*	106		
Low (−/+)	60 (57)	1	
High (++/+++)	46 (43)	4.73	1.71–13.08 (*P*=0.003)
			
*CTTN*	106		
Low (<13%)	55 (52)	1	
High (>13%)	51 (48)	13.14	3.04–56.87 (*P*=0.0006)
			
*Lymph nodes*	106		
Negative (cN−)	67 (63)	1	
Positive (cN+)	39 (37)	3.16	1.29–7.75 (*P*=0.012)
			
*Treatment*	106		
Surgery	42 (40)	1	
Radiotherapy	16 (15)	4.49	1.51–13.41 (*P*=0.007)
Both	48 (45)	1.32	0.44–3.94 (*P*=0.62)

Abbreviations: CI=confidence interval; DSM=disease specific mortality; HR=hazard ratio; N pos.=number of positive cases.

Age, gender, T-status and grade were not significant and not included in this table.

**Table 4A:** Multivariate Cox regression for CCND1, CTTN, FADD and lymph node metastasis to DSM

**Cox regression**		**Hazard ratio for DSM**	
**multivariate**	***N* (%)**	**HR**	**95% CI**
*CCND1*	106		
Low (<23%)	52 (49)	1	
High (>23%)	54 (51)	1.28	0.38–4.24 (*P*=0.69)
			
*FADD*	106		
Low (−/+)	60 (57)	1	
High (++/+++)	46 (43)	1.96	0.57–6.68 (*P*=0.29)
			
*CTTN*	106		
Low (<13%)	55 (52)	1	
High (>13%)	51 (48)	8.46	1.63–43.88 (*P*=0.011)
			
*Lymph nodes*	106		
Negative (cN−)	67 (63)	1	
Positive (cN+)	39 (37)	3.96	1.30–12.06 (*P*=0.015)
			
*Treatment*	106		
Surgery	42 (40)	1	
Radiotherapy	16 (15)	2.72	0.75–9.86 (*P*=0.13)
Both	48 (45)	0.75	1.50–13.31 (*P*=0.67)

Abbreviations: CI=confidence interval; DSM=disease specific mortality; HR=hazard ratio; N pos.=number of positive cases.

**Table 4B:** Multivariate Cox regression for CTTN and lymph node metastasis to DSM

**Cox regression**		**Hazard ratio for DSM**	
**multivariate**	***N* (%)**	**HR**	**95% CI**
*CTTN*	106		
Low (<13%)	55 (52)	1	
High (>13%)	51 (48)	15.96	3.55–71.73 (*P*=0.0003)
			
*Lymph nodes*	106		
Negative (cN−)	67 (63)	1	
Positive (cN+)	39 (37)	4.03	1.57–10.35 (*P*=0.004)

Abbreviations: CI=confidence interval; DSM=disease specific mortality; HR=hazard ratio; N pos.=number of positive cases.
